# Circular RNA ciR-02852: A novel physiological inhibitor of Porcine ovarian granulosa cell functions

**DOI:** 10.1007/s11033-026-11539-x

**Published:** 2026-02-09

**Authors:** Zuzana Fabová, Barbora Loncová, Abdel Halim Harrath, Anouar Feriani, Alexander V. Sirotkin

**Affiliations:** 1 Faculty of Natural Sciences and Informatics, Constantine the Philosopher University in Nitra, Nitra, 949 74 Slovakia; 2 Department of Zoology, College of Science, King Saud University, Riyadh, 11451 Saudi Arabia; 3 Laboratory of Biotechnology and Biomonitoring of the Environment and Oasis Ecosystems, University of Gafsa, Gafsa, 2112 Tunisia; 4 Faculty of Natural Sciences and Informatics, Constantine the Philosopher University, Tr. A. Hlinku 1, Nitra, 949 74 Slovakia

**Keywords:** CircRNA, CiR-02852, Ovary, Granulosa cells, Apoptosis, Steroidogenesis

## Abstract

**Background:**

Circular RNAs (circRNAs) are stable epigenetic regulators of various biological processes, yet the functional role of ciR-02852 remains unknown. This study aimed to characterize the influence of ciR-02852 on the fundamental functions of porcine ovarian granulosa cells.

**Methods:**

Cells were transfected with either a ciR-02852-overexpressing vector or an shRNA ciR-02852-knockdown vector. We assessed cell viability, proliferation markers (PCNA, cyclin B1), apoptosis markers (Bax, caspase-3, and DNA fragmentation), and the secretion of steroid (progesterone, testosterone, estradiol) and peptide (IGF-I, oxytocin) hormones via RT-qPCR, immunocytochemistry, TUNEL, and ELISA.

**Results:**

Overexpression of ciR-02852 significantly reduced cell viability and the expression of PCNA and cyclin B1. Conversely, it stimulated both cytoplasmic and nuclear apoptosis, as evidenced by increased Bax, caspase-3, and DNA fragmentation. Furthermore, ciR-02852 overexpression inhibited the release of all measured hormones. Silencing of ciR-02852 via shRNA yielded the opposite effects, promoting proliferation and hormone output while suppressing apoptosis.

**Conclusion:**

These findings demonstrate for the first time that ciR-02852 acts as a potent physiological inhibitor of porcine ovarian cell functions. Our results indicate that ciR-02852 could be a multi-target regulator of folliculogenesis and hormone production, highlighting its potential as a diagnostic marker or therapeutic target for managing reproductive disorders and improving animal production.

## Introduction

Epigenetic elements, including small noncoding RNAs, could be powerful regulators of reproductive processes at the level of transcription, translation, and the expression of all known genes involved. Such RNA interference could be potentially applied in reproductive biology, biotechnology, assisted reproduction, animal production, and the treatment of reproductive disorders [1–4]. However, the wide application of various small noncoding RNAs is limited by their instability and rapid breakdown by nucleases [2].

One class of small RNAs, circular RNAs (circRNAs), possesses a unique closed-loop structure lacking polyadenylated tails, which renders them highly stable and resistant to exonuclease degradation. CircRNAs regulate gene splicing and transcription and can function as sponges for other small RNAs (miRNAs), as well as sponges and modulators of proteins and protein-coding RNAs [5, 6]. The variation in the expression of some circRNAs during specific phases of ovarian folliculogenesis, oogenesis, embryogenesis, as well as reproductive aging, ovarian insufficiency, cancer, and polycystic ovarian syndrome provides circumstantial evidence of the involvement of circRNAs in the regulation of various female reproductive events and disorders [4, 7–9].

Furthermore, there is direct proof regarding the effects of some circRNAs on the proliferation of cancer [10] and healthy [11] ovarian cells and on the apoptosis of cancer [12] and healthy [6, 11] ovarian cells. Finally, the ability of ciR-00596 and ciR-00646 to influence the release of hormones (progesterone, testosterone, estradiol, insulin-like growth factor I (IGF-I), and oxytocin) by cultured healthy porcine ovarian granulosa cells has been reported [11].

The expression of ciR-02852 in the porcine ovary has been documented [13], but its function remains to be determined. The present study sought to examine the influence of both upregulation and downregulation of ciR-02852 on fundamental ovarian functions. For this purpose, we transfected porcine granulosa cells with a vector overexpressing ciR-02852 and an shRNA vector designed to suppress ciR-02852. Following this, we assessed the relative expression levels of ciR-02852, cell viability; proliferation (measured by the accumulation of PCNA and cyclin B1, which are markers of S- and G- phases of mitosis); cytoplasmic (assessed via the accumulation of Bax and caspase-3) and nuclear (DNA fragmentation) apoptosis; and the release of progesterone, testosterone, estradiol, IGF-I, and oxytocin. The key role of these processes in the control of ovarian folliculogenesis, follicular selection, growth, ovulation, oogenesis, embryogenesis, and reproductive lifespan is well established [3, 14].

## Materials and methods

### Construction of knockdown and overexpression plasmids

A short hairpin RNA (shRNA) designed to target the back-splice junction (BSJ) region of ciR-02852 (which was termed sh-ciR-02852) was created and subsequently inserted into the pGPU6/green fluorescent protein (GFP)/Neo vector (GenePharma, Shanghai, China). A negative control shRNA (sh-NC) was also developed using the same vector; however, this version contained sequences that did not match the BSJ region. The complete sequence of ciR-02852 was placed into the pEX-3 (pGCMV/MCS/Neo) vector to create the overexpression vehicle. A pEX-3 vector that was devoid of an insert was used as the negative control. The sequences for all the resulting constructs (refer to Tables 1 and 2) were sourced from GenePharma. The control groups incorporated cells that were either left nontransfected or were transfected with negative controls (NCs). The effectiveness of both the gene suppression and overexpression of ciR-02852 was evaluated using reverse transcription‒quantitative polymerase chain reaction (RT‒qPCR).

**Table 1:** .The sequences of ShRNA vectors

shRNA expression vector	Sequence	
sh-ssc-ciR-02852	Sense	5′-CACCGTGGTGTATGCCTCAGTGGTATTCAAGAGATACCACTGAGGCATACACCACTTTTTTG-3′
	Antisense	5′-GATCCAAAAAAGTGGTGTATGCCTCAGTGGTATCTCTTGAATACCACTGAGGCATACACCAC-3′
shRNA negative control	Sense	5′-CACCGTTCTCCGAACGTGTCACGTTTCAAGAGAACGTGACACGTTCGGAGAATTTTTTG-3′
	Antisense	5′-GATCCAAAAAATTCTCCGAACGTGTCACGTTCTCTTGAAACGTGACACGTTCGGAGAAC-3′

**Table 2:** The sequence of overexpression vector

Overexpression vector	Sequence
ssc-ciR-02852	5′-TGCCTCAGTGGTAACAAACCCAACTAGTATTCATGAGGATGTGGGTTCAATCCCCAGCCTTG TTCAGTGGGTTAAAGGATCTGGCAGTGCCATAAGCTGTGGTGTA-3′

### Preparation, processing, culture and transfection of ovarian granulosa cells

A total of twenty ovaries from Landrace gilts in the prepubertal stage (aged 6–8 months) were acquired from the Chovmat F.U. slaughterhouse in Rastislavice, Slovakia. Ovaries and granulosa cells were isolated, processed and cultured in DMEM/F12 1:1 medium (BioWhittaker™; Lonza, Verviers, Belgium) supplemented with 10% fetal bovine serum (BioWhittaker™) and 1% antibiotic-antimycotic solution (Sigma‒Aldrich, St. Louis, MO, USA) as it was described previously [11, 15]. Following initial preculture during 2–3 days, the cells were subjected to transfection with a knockdown plasmid (sh-ciR-02852) or an overexpression plasmid (ciR-02852) alongside their respective negative controls (NCs) by using Lipofectamine^®^ RNAiMAX transfection reagent (Invitrogen, Carlsbad, CA, USA). The control groups encompassed nontransfected cells and cells transfected with NC plasmids. 48 h after transfection, the medium was used for ELISA analysis, while cells were used for immunocytochemistry, Trypan blue exclusion tests, TUNEL assay and RT-qPCR.

### Cell viability test, TUNEL assay and immunocytochemical analysis

Cell viability was determined using a Trypan blue (0.4%) exclusion test according to standard method described previously [16, 17] in our modification [11, 15]. DNA fragmentation within the cell culture was evaluated via the TUNEL assay (HT TiterTACS™ Apoptosis Detection Kit; Trevigen, Gaithersburg, MD, USA) and ELISA reader (Thermo Fisher Scientific, Inc.) following the manufacturer’s protocol. Immunocytochemistry to visualize the presence and quantification of proteins associated with cell proliferation (PCNA and cyclin B1) and apoptosis (Bax and caspase-3) was performed by using primary mouse monoclonal antibodies (Santa Cruz Biotechnology, Inc., CA, USA), and secondary anti-mouse IgG labelled with horseradish peroxidase (Santa Cruz Biotechnology, Inc.) or CruzFluor™ (CFL594) (Sigma‒Aldrich,) as described previously [11, 15]. Quantification was achieved by calculating the percentage of stained cells compared to the total cell population.

### RT‒qPCR

Total RNA (totally 800 ng) was extracted from the transfected granulosa cells using the TRIzol reagent (Invitrogen) following the manufacturer’s protocol. The integrity and concentration of the extracted RNA were then evaluated via a UV spectrophotometer (Bio-Rad, Inc., Hercules, CA, USA), thereby confirming high purity and yield, which are essential for subsequent applications. The extracted RNA was subsequently reverse transcribed into complementary DNA (cDNA) via the PrimeScript RT reagent kit (Takara Bio, Inc., Shiga, Japan) according to the manufacturer’s protocol. qPCR was performed via the SYBR Premix Ex Taq II Kit (TaKaRa, Dalian, China) using an ABI 7500 Fast Real-Time PCR System (Thermo Fisher Scientific, Inc.). The qPCR amplification protocol was optimized for both accuracy and efficiency, commencing with initial denaturation at 95 °C for 7 min to activate Taq DNA polymerase, followed by 40 cycles of thermal cycling: denaturation at 95 °C for 10 s, annealing at 60 °C for 10 s, and extension at 72 °C for 10 s. The housekeeping gene GAPDH served as the internal reference to normalize expression levels across different samples, and relative gene expression was calculated via the commonly used $2^{–\Delta\Delta Ct}$ method [18]. The primer sequences (Table 3) were designed and synthesized by GenePharma. Each RNA sample was analyzed in triplicate to minimize experimental variation, and the resulting average values were calculated to provide a robust measure of gene expression.

**Table 3:** The sequences of gene primers for RT-qPCR

Gene symbol	Reference (publication or GenBank accession number)	Primer sequence	
ssc-ciR-02852	Liang et al., 2017	Forward	5′-AGTGGGTTAAAGGATCTGGCA-3′
	Liang et al., 2017	Reverse	5′-GGTTTGTTACCACTGAGGCAT-3′
GAPDH	U48832	Forward	5′-GAAGGTGAAGGTCGGAGT-3′
	U48832	Reverse	5′-AAGATGGTGATGGGATTTC-3′

### Enzyme-linked immunosorbent assay (ELISA)

The concentrations of key steroid hormones—progesterone, testosterone, 17beta-estradiol, as well as insulin-like growth factor I (IGF-I) and oxytocin—were quantified in aliquots of 25 µL or 100 µL of the granulosa cell incubation medium via enzyme-linked immunosorbent assays (ELISAs) in accordance with the manufacturer’s protocol. The ELISA kits for progesterone (Cat. No. FR E-2500), testosterone (Cat. No. AA E-1300), 17beta-estradiol (Cat. No. FR E-2000) and IGF-I (Cat. No. ME E-0500) were obtained from LDN Immunoassays and Services (Nordhorn, Germany). The oxytocin ELISA kit (Cat. No. ab133050) was acquired from Abcam (Cambridge, UK). The detailed assay characteristics are provided in Table 4.

**Table 4:** Characteristics of the immunoassays used in experiments

Substance assayed	Specificity of assay (cross-reactivity of antiserum)	Sensitivity of assay (ng/ml)	Coefficient of variation (%)	
			Intra-assay	Inter-assay
Progesterone	≤1.1% with 11-desoxycorticosterone, ≤0.35% with pregnenolone, ≤0.30% 17α-OH with progesterone, ≤0.20% with corticosterone, ˂0.10% with estriol, 17β-estradiol, testosterone, cortisone and 11-desoxycortisol, ˂0.02% with DHEA-S and cortisol	0.045	5.4	5.59
Testosterone	≤3.3% with 11β-hydroxytestosterone and 19-nortestosterone, ≤0.9% with androstenedione, ≤0.8% with 5α-dihydrotestosterone, and ˂0.1% with 17α-methyltestosterone, epitestosterone, estradiol, progesterone, cortisol, estrone, and danazol	0.083	4.16	4.73
***17β*** -estradiol	≤9.5% with fulvestrant, ≤4.2% with estrone, ≤3.8% with E2-3-glucuronide, ≤3.6% with E2-3-sulphate, ≤0.4% with estriol, ˂0.1% with androstenedione, 17-hydroxyprogesterone, corticosterone, pregnenolone, E2-17-glucuronide, progesterone, and testosterone	0.0062	6.4	4.5
IGF-I	100% with IGF-I, ≤3.3% with insulin, and 1.02% with IGF-II	9.75	7.39	12.63
Oxytocin	100% with oxytocin, 7.5% with arg8-vasotocin, 7.0% with mesotocin, and ˂0.2% with ser4,lle8-oxytocin, TRH, somatostatin, met-enkephalin, VIP, lys8-vasopressin and arg8-vasopressin	0.015	12.6	11.8

### Statistical analysis

The data presented in this study represent the mean values obtained from three independent experiments, using separate sets of granulosa cells isolated from at least six different ovaries. Four culture wells containing ovarian granulosa cells were utilized for each experimental condition. The cell viability was determined by counting a minimum of 100 cells per well, while TUNEL and immunocytochemical analysis was used to ascertain the percentage of antigen-positive cells from at least 1,000 cells per well. For the ELISAs, nonspecific background values (less than 10% of the total readings) were subtracted from the measured values of cell-conditioned media. The secretion rates of hormones and growth factors were normalized to 10^6^ viable cells per day. For statistical analysis, the values of technical replicates (4 wells used within each experiment for each condition) were averaged to generate a single data point for each biological replicate (*n* = 3). Statistical analyses were performed with the Shapiro‒Wilk normality test to confirm the data distribution, followed by Student’s t test for pairwise comparisons and one-way ANOVA with Tukey’s post hoc test to assess differences across multiple groups (e.g., NC vs. Overexpression vs. Knockdown) to determine overall significance before post-hoc testing. All analyses were conducted via SigmaPlot 11.0 (Systat Software, GmbH, Erkrath, Germany). Statistical significance was defined at *P* < 0.05, with differences considered significant if they met this threshold.

## Results

### Transfection efficiency

**Fig. 1:**
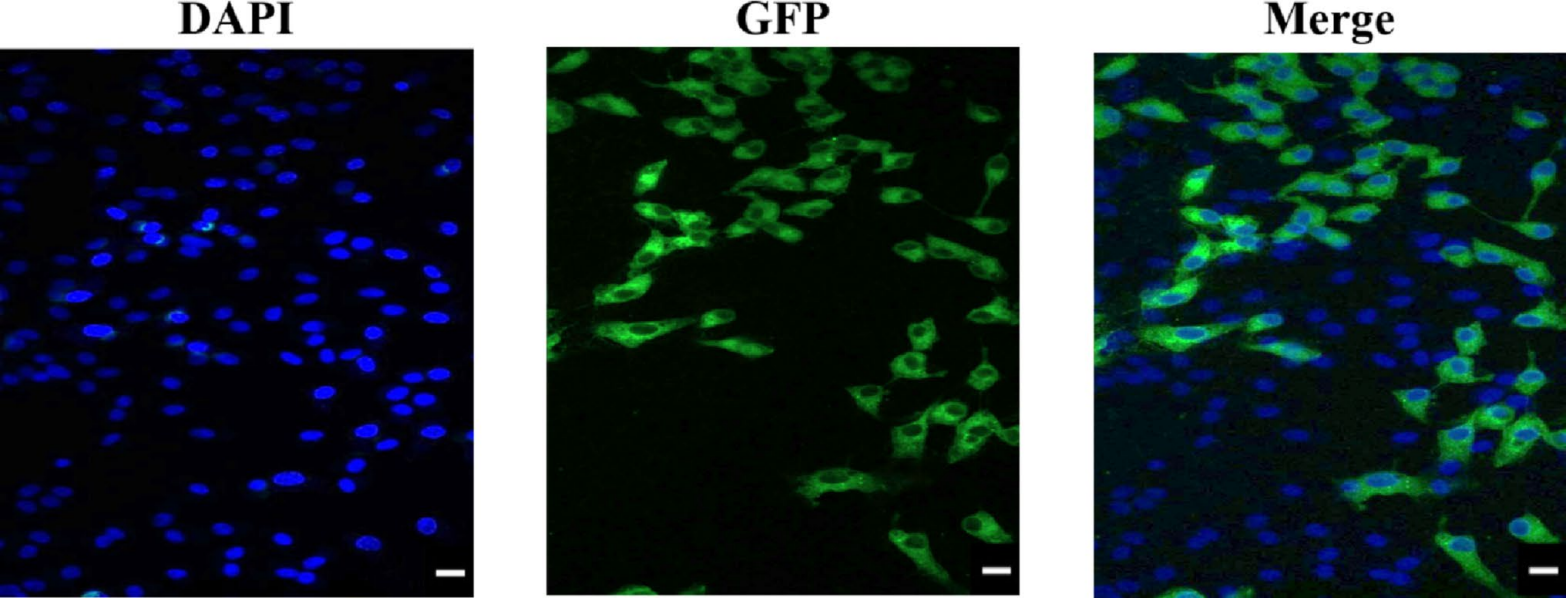
Transfection efficiency of porcine ovarian granulosa cells. Representative images showing cells displaying green fluorescent protein (GFP) expression 48 h after transfection with the pGPU6/GFP/Neo-sh-NC plasmid construct. Original magnification: 200x

**Fig. 2:**
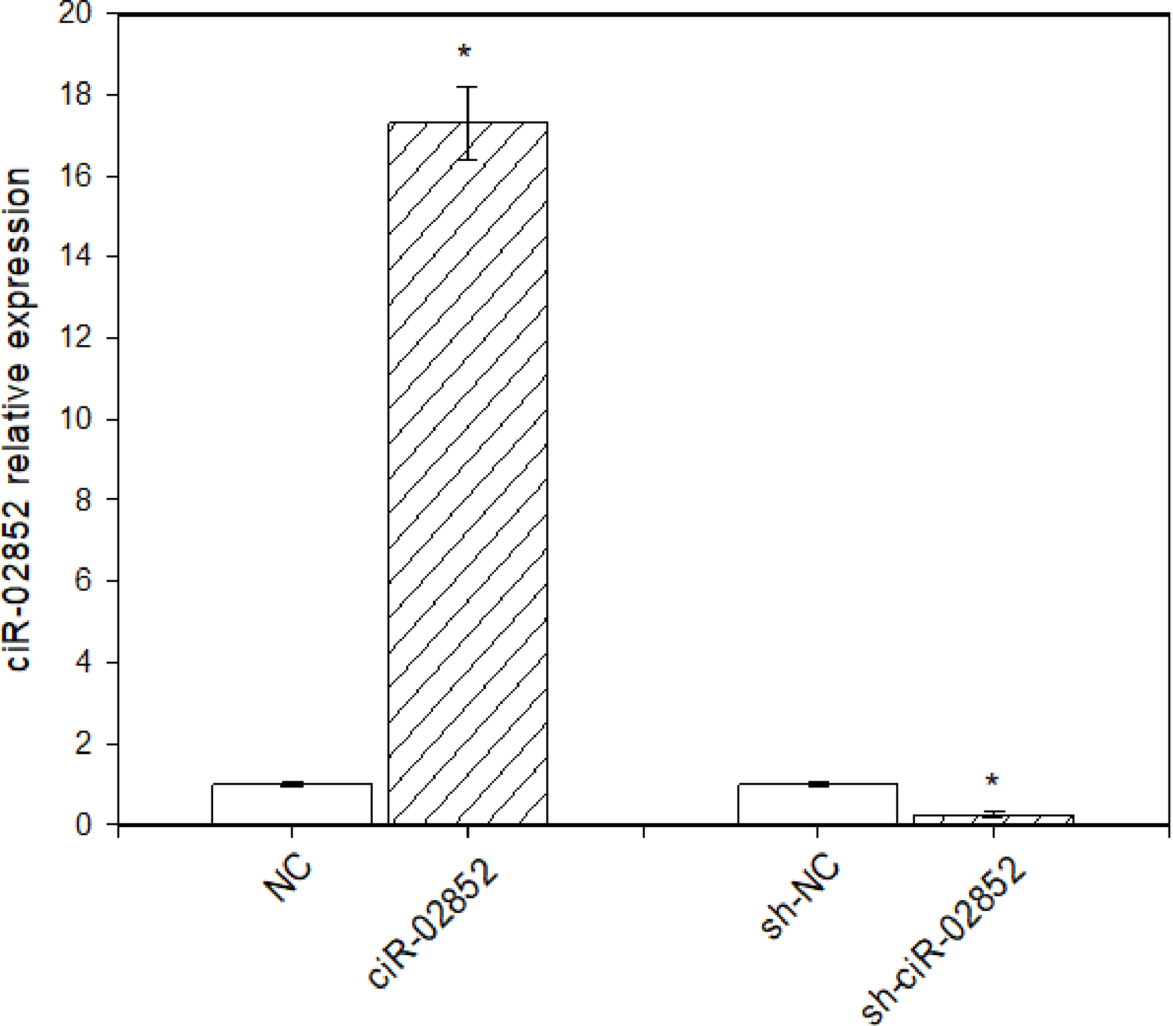
Relative expression levels of ciR-02852 in porcine ovarian granulosa cells. The relative expression was measured via RT–qPCR in nontransfected cells (Control), cells transfected with negative control vectors (NC), and cells transfected with either the ciR-02852-overexpressing vector (Overexpression) or the shRNA knockdown vector (sh-ciR-02852). Data are presented as the mean ± SEM. * denotes significant differences (*P* < 0.05) with negative control

In this study, porcine ovarian granulosa cells were transfected with the ciR-02852-overexpressing vector and the shRNA knockdown vector for ciR-02852 {sh-ciR-02852}, in addition to their respective negative controls (NCs). Transfection efficiency was shown via two methods: (1) more than 83% of cells displayed GFP inserted into the pGPU6/GFP/Neo-sh-NC plasmid construct (Fig. 1), and (2) RT–qPCR was used to verify that ciR-02852 integration and expression levels rose in granulosa cells after ciR-02852 overexpression but fell after transfection with sh-ciR-02852 (Fig. 2).

### Effects of ciR-02852 overexpression and knockdown on Porcine ovarian granulosa cells

**Fig. 3:**
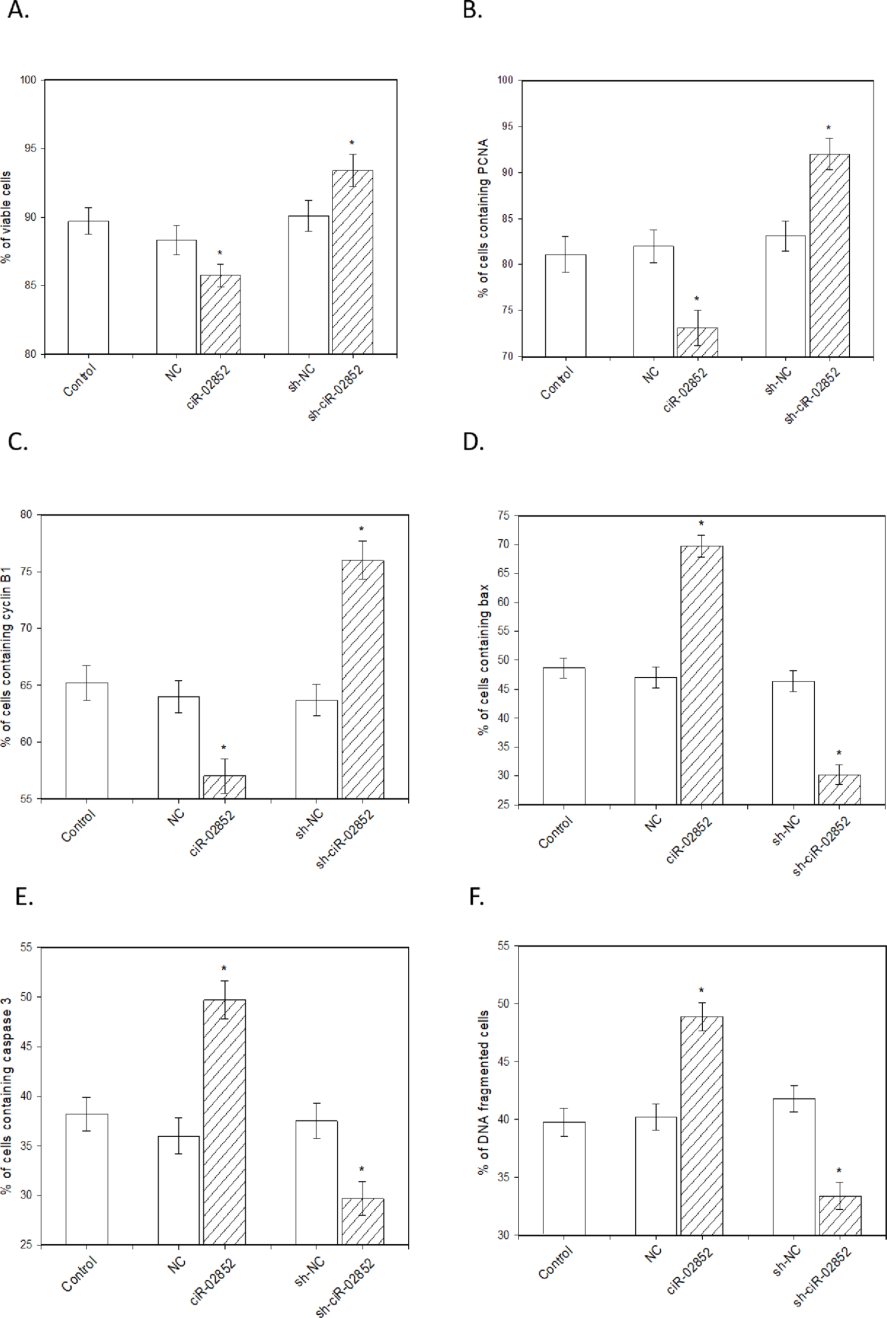
Influence of ciR-02852 overexpression and knockdown on porcine ovarian cell viability, proliferation, and apoptosis. (A) Cell viability determined by the Trypan blue exclusion test. (B) Percentage of cells positive for PCNA (S-phase marker). (C) Percentage of cells positive for cyclin B1 (G2-phase marker). (D) Percentage of cells positive for the proapoptotic protein Bax. (E) Percentage of cells positive for caspase-3. (F) Rate of nuclear apoptosis measured by DNA fragmentation (TUNEL assay). Data represent mean values ± SEM. * denotes significant differences (*P* < 0.05) with negative control

**Fig. 4:**
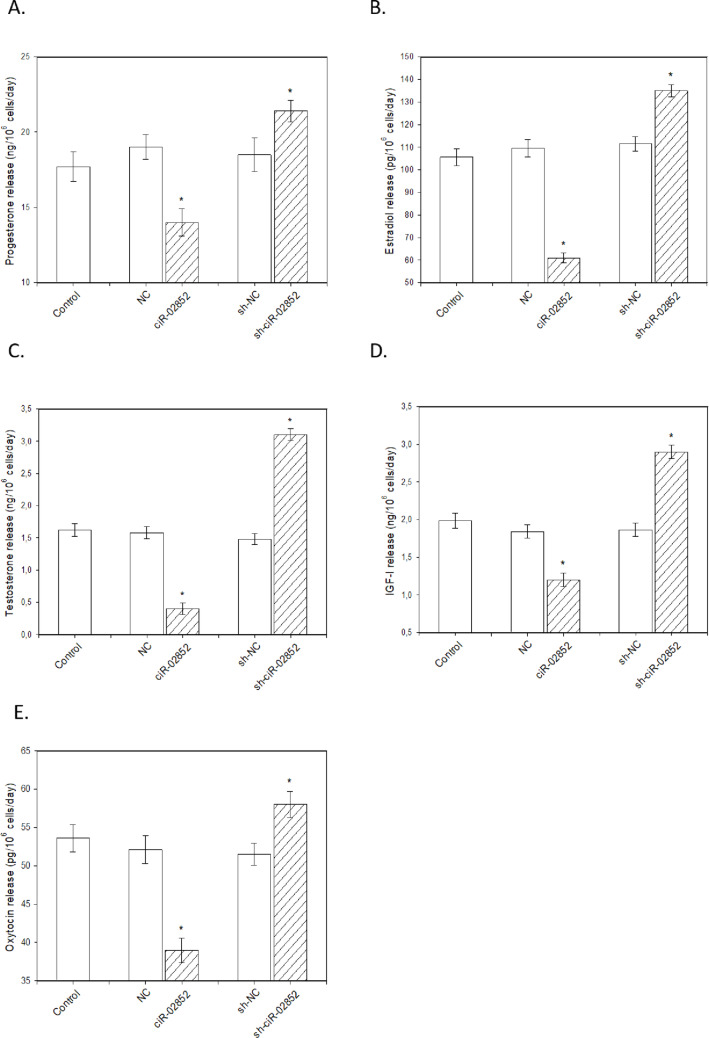
Influence of ciR-02852 overexpression and knockdown on the release of steroid and peptide hormones by porcine ovarian granulosa cells. Concentrations of (A) progesterone, (B) estradiol, (C) testosterone, (D) insulin-like growth factor I (IGF-I), and (E) oxytocin were measured in the culture medium via ELISA. Hormone concentrations were normalized to 10^6^ viable cells per day. Data are presented as the mean ± SEM. * denotes significant differences (*P* < 0.05) with negative control

The overexpression of ciR-02852 led to a reduction in cell viability (Fig. 3A) and in the accumulation of PCNA (Fig. 3B) and cyclin B (Fig. 3C) positive cells. Furthermore, the number of apoptotic cells increased, as evidenced by the proportions of Bax- (Fig. 3D) and caspase-3- (Fig. 3E) positive cells and cells with DNA fragmentation (Fig. 3F). Moreover, ciR-02852 overexpression hindered the release of progesterone (Fig. 4A), estradiol (Fig. 4B), testosterone (Fig. 4C), IGF-I (Fig. 4D), and oxytocin (Fig. 4E). Conversely, sh-ciR-02852 expression enhanced cell viability (Fig. 3A) and the accumulation of PCNA (Fig. 3B) and cyclin B (Fig. 3C). Sh-ciR-02852 also diminished the accumulation of the proapoptotic peptides Bax (Fig. 3D) and caspase-3 (Fig. 3F) and the number of TUNEL-positive cells (Fig. 3G). Furthermore, sh-ciR-02852 was found to promote the release of the investigated steroid (Figs. 4A-C) and peptide hormones (Figs. 4D-E).

## Discussion

The cells utilized in these experiments were viable and active, a fact confirmed by their ability to form a cellular monolayer in culture, the presence of molecules associated with proliferation such as PCNA and cyclin B1, their capacity to exclude Trypan blue, and their secretion of steroid and peptide hormones. Moreover, the inclusion of the fluorescent marker GFP and RT–qPCR analysis of ciR-02852 expression following the transfection of cells with the corresponding agents, which successfully induced both elevated and reduced levels of ciR-02852 expression, verified the efficacy and reliability of the method employed for cell transfection and the manipulation of circRNA expression.

The expression of several circRNAs, including ciR-02852 in mammalian [7] and porcine [13] ovaries, as well as alterations in their expression linked to the reproductive state, has been documented. However, the precise function of the majority of circRNAs in the governance of reproductive processes remains unknown. Our research is the initial study to demonstrate that ciR-02852 can play a critical part in the control of fundamental ovarian cell functions. In the present study, transfecting porcine granulosa cells with the ciR-02852 overexpression vector resulted in a significant decrease in the accumulation of proliferation-related proteins, whereas the silencing of this circRNA with shRNA caused an increase. These proteins included PCNA (an endogenous promoter and marker of the S phase of mitosis) [19] and cyclin B1 (which manages the transition from the G2- to the S phase of the cell cycle) [20]. These findings suggest that ciR-02852 restricts mitosis at both the G2- and S- phases of the cell cycle by modifying these regulators and markers. Cell proliferation is the primary mechanism involved in ovarian folliculogenesis, follicle selection, and oogenesis [21]. The ability of ciR-02852 to inhibit ovarian cell proliferation can indicate its function as a physiological suppressor of these proliferation-related reproductive processes, although this hypothesis requires validation by further in-vivo experiments.

The present study demonstrated that reduced ovarian cell proliferation may be associated with increased apoptosis. Apoptosis is a crucial process involved in oogenesis, folliculogenesis, oocyte selection, and atresia [22–24]. In the present study, the overexpression of ciR-02852 promoted the accumulation of Bax and caspase-3, while the silencing of ciR-02852 prevented their accumulation. Both Bax and caspase-3 are known as markers and promoters of cytoplasmic/mitochondrial apoptosis [25, 26]. Furthermore, changes in ciR-02852 induced similar changes in histone-linked DNA fragmentation, whereas the silencing of this circRNA with shRNA caused a decrease in DNA fragmentation. Such DNA fragmentation is the main feature of nuclear apoptosis in granulosa cells [27]. The functional interrelationships between these collaborating processes—cytoplasmic and nuclear apoptosis—may be postulated. Caspases could be promoters of DNA fragmentation [28]. The present observations suggest that ciR-02852 could be a physiological trigger of both cytoplasmic and nuclear apoptosis in ovarian cells. It is not to be excluded, that ciR-02852 could be a regulator of the apoptosis-related reproductive events listed above, although this hypothesis requires experimental confirmation too.

In the present experiments, overexpression of ciR-02852 decreased cell viability, but the silencing of this circRNA caused an increase. Cell viability, which is governed by the balance between cell proliferation and apoptosis, determines the selection of an ovarian follicle—leading either to its growth and development or to atresia and subsequent degeneration [14, 23, 24]. These observations suggest that ciR-02852 can lower ovarian cell viability, possibly through a decreased proliferation/apoptosis ratio. These effects indicate that ciR-02852 could play a crucial role in the induction of follicular atresia and selection, although ciR-02852 influence on folliculogenesis remain to be studied yet. CiR-02852 may influence ovarian cell viability via the release of steroids and peptide hormones, which are considered as extracellular regulators of ovarian cell viability, proliferation, and apoptosis [14]. For example, the influence of progesterone [29], estradiol [30], testosterone [31], IGF-I [32] and oxytocin [33] on ovarian cells proliferation, apoptosis and viability has been documented. On the other hand, vice versa, viability of ovarian cells can play a crucial role in control of release of ovarian hormones [23, 34].

In the present study, ciR-02852 dampened the release of all measured ovarian hormones, including progesterone, testosterone, estradiol, IGF-I, and oxytocin. Conversely, the silencing of ciR-02852 enhanced the release of all these hormones. The ability of ciR-02852 to reduce the release of these hormones suggests its potential involvement in regulating hormone-dependent ovarian processes. For example, all these hormones are considered regulators (mainly promoters) of ovarian cell proliferation, folliculogenesis, and luteinization and inhibitors of ovarian cell apoptosis and follicular atresia [14]. It is possible that some hormonal ciR-02852-induced changes are direct, whereas others are indirect. For example, progesterone is considered a precursor to testosterone, which in turn is a precursor to estradiol [35]. Thus, ciR-02852-induced diminished release of testosterone and estradiol may be explained by the downregulation of the synthesis of their precursors. Furthermore, ciR-02852 may inhibit ovarian steroidogenesis via the suppression of IGF-I and oxytocin, whose stimulatory influence on the release of ovarian steroid hormones is well documented [14]. In contrast, the ability of some steroid hormones to promote oxytocin and IGF-I release, as well as the reciprocal stimulation of ovarian oxytocin and IGF-I release, has been reported [36]. Understanding the hierarchical interrelationships between ovarian hormones and other ovarian functions requires further study, but this is the first evidence that ciR-02852 could be a physiological inhibitor of the release of steroid and peptide hormones and, maybe, of hormone-dependent events in ovarian cells.

## Conclusion, study Limitations, and possible directions for future studies

The present findings demonstrated for the first time that ciR-02852, which is present in the porcine ovary [13], could be a potent physiological suppressor of core ovarian cell functions: it decreases cell proliferation (likely at both the M- and G- phases of mitosis), cell viability, the release of ovarian steroid and peptide hormones and stimulates cytoplasmic and nuclear apoptosis. Therefore, the present novel results broadened the list of possible regulators of female reproductive processes. Moreover, to our knowledge, this is the first evidence of the physiological role of ciR-02852. Finally, the observed influence of ciR-02852 on regulators of several basic cellular events demonstrates multiple targets of ciR-02852 and its possible involvement in control of physiological processes others than reproduction.

The ability of ciR-02852 and its inhibitor to significantly alter all the measured ovarian parameters, as well as potentially influencing the low degradation of circRNA (as noted previously), could indicate the potential practical use of ciR-02852 regulators. Its presence in the ovary and its suppressive function described here suggest that ciR-02852 might be potentially useful for the forecasting of the reproductive state and its dysfunctions, including infertility. Furthermore, the promotion of ciR-02852 might be useful in farm animal production for temporarily blocking the ovarian cycle for synchronization or in clinical use for the suppression of ovarian cell functions in cases of ovarian cancer. On the other hand, blocking ciR-02852 could be used for stimulating ovarian functions and treating ovarian insufficiency and infertility. The diagnostic and therapeutic potential of ciR-02852 could be an area worth further in-vitro and in-vivo study.

Nevertheless, it is to note, that the current study has the preliminary, descriptive and exploratory character. Further research is needed to address the possible limitations of the present study. The present results obtained during in vitro experiments should be validated by in vivo studies. The direct experimental evidence for ciR-02852 influence on ovarian folliculogenesis and fertility, as well as for its therapeutic potential, which have been hypothesized above, is missing yet. Identification of the mediators of ciR-02852 and their possible functional interrelationships mentioned above requires further clarification. In addition to the hormones and proteins regulating the cell cycle and apoptosis studied here, circRNAs can influence ovarian functions by affecting microRNAs [5, 6, 10, 12, 13, 37]. Exploring the interrelationships between ciR-02852 and other small RNAs requires further investigation. Finally, if the speculations about the applicability of ciR-02852 in controlling reproductive processes and treating their disorders formulated above are confirmed by further in vivo studies, exploring strategies for the delivery and application of ciR-02852 regulators—either alone or in combination with traditional pharmaceutical treatments—could contribute to animal production and medicine.

## Data Availability

The primary data can be provided upon request.
